# Assessment of intestinal and blood protozoan infections among pregnant women visiting ante-natal care at Tafo Hospital, Ghana

**DOI:** 10.1016/j.heliyon.2022.e09968

**Published:** 2022-07-20

**Authors:** Emmanuel Amaniampong Atakorah, Bright Oppong Afranie, Kwabena Darko Addy, Ama Darkoaa Sarfo, Bright Afranie Okyere

**Affiliations:** a Department of Clinical Microbiology, School of Medicine and Dentistry, Kwame Nkrumah University of Science and Technology, Kumasi, Ghana; b Laboratory Department, Tafo Government Hospital, Tafo, Kumasi, Ghana; c Department of Molecular Medicine, School of Medicine and Dentistry. Kwame Nkrumah University of Science and Technology, Kumasi, Ghana; d Department of Medical Laboratory Technology, School of Allied Health Sciences, University of Cape Coast, Cape Coast, Ghana; e Pediatric Infectious Disease Unit, Child Health Directorate, Komfo Anokye Teaching Hospital, Kumasi Ghana; f Department of Pharmaceutics, Faculty of Pharmacy and Pharmaceutical Sciences, Kwame Nkrumah University of Science and Technology, Kumasi, Ghana

**Keywords:** Intestinal protozoans, Pregnant women, *Toxoplasma gondii* antibodies, Malaria infection

## Abstract

**Introduction:**

Intestinal and blood protozoans cause morbidity and mortality in both pregnant women and developing foetuses worldwide. It constitutes a major health problem in many tropical areas in Africa. This study assessed the prevalence of intestinal and blood protozoans’ parasitic load and their risk factors among pregnant women visiting antenatal care at Tafo Hospital, Ghana from November 2016 to January 2017.

**Method:**

A pilot cross-sectional study was conducted among consented pregnant women who visited antenatal care at Tafo Government Hospital, Kumasi Ghana. Structured questionnaires were administered to obtain socio-demographic data, knowledge on protozoan infections, and their risk factors among study participants. A stool sample was obtained from each participant for the microscopic examination of the intestinal protozoa. Venous blood was taken from participants for the detection of Plasmodium and *Toxoplasma gondii* infections. Wet mount and the faecal protozoan concentrated method were done for the identification of intestinal parasites. Blood films and serological examination for malaria rapid diagnostic tests (RDT) were done for identification of Plasmodium parasites while an Enzyme-linked immunosorbent assay (ELISA) was used for detecting the antibodies of *T. gondii* among participants. Data were analyzed using statistical packages for the social sciences (SPSS).

**Results:**

The mean age of the study participants was 27.83, and ranges from 18 to 40 years. The majority of the participants (82.2%) had never experienced stillbirth nor spontaneous abortion. Intestinal parasites were found in 36.7% of participants. *Giardia lamblia* (28.1%), *Cryptosporidium parvum* (5.3%), and *Entamoeba histolytica/dispar* (3.3%) were among the intestinal protozoans detected*. T. gondii* antibodies were detected by high levels of immunoglobulins, resulting in IgG (48.0%) and IgM (11.3%) being found among participants, with 7.3% testing positive for both IgM and IgG. The prevalence of malaria infection among the study participants was 2.7%. The consumption of raw or cooked vegetables had significant influence on their intestinal and blood protozoan infections status (p = 0.004) (OR = 0.32, CI = 0.12–0.86). There was a significant association between Hb levels and malaria (p = 0.014) and that of intestinal protozoans (p = 0.035).

**Conclusion:**

The prevalence of intestinal protozoans and blood protozoans such as *T*. *gondii* were high and therefore effective measures should be put in place to reduce the infectivity. Environmental hygiene should be improved and education by relevant agencies should be intensified on the possible transmission of intestinal and blood parasite infections given the possible role of these infections in adverse pregnancy outcomes.

## 1 Introduction

Protozoan parasites are the most common cause of infection, with significant rates of morbidity and mortality, especially in impoverished countries with inadequate resources [1, 2]. The prevalence of these neglected parasitic infections is almost 50% in developed countries and is as high as 90% in developing countries with the highest burden of infections in Sub-Saharan Africa [1, 3].

Most diarrheal diseases have been associated with intestinal protozoans such as giardiasis, amoebiasis, and cryptosporidiosis. These parasitic protozoans cause morbidity that burdens the health of people [1, 4].

Intestinal parasitic infections (IPIs), such as soil-transmitted helminth (STH) and protozoan infections, are well-known drivers of morbidity and illness in underserved groups. There are between 450 and 840 million cases worldwide, with 95 percent of them happening in developing countries [5]. Data from previous studies in Ghana showed that intestinal parasitic infections among pregnant women ranges from 41.2% [6] to 49.6% [7]. Moreover, blood protozoan infections such as malaria and toxoplasmosis infections have great economic implications as funds used for the treatment of patients and procurement of drugs can be channeled to another sector. Disease caused by intestinal and blood protozoans has been identified as one of the leading causes of morbidity and mortality worldwide [8, 9]. Plasmodium, the protozoan with the largest illness burden in Africa, can influence the evolution of other parasitic diseases in a good or negative way without necessarily being impacted [10]. In Ghana*, Plasmodium falciparum* infection is the most prevalent cause of consultation and hospitalization for a febrile illness, and the number of clinical cases is on the rise [11]. Malaria among pregnant women accounts for around 14% of Out Patient Department (OPD) attendance, 11% of admissions, and 9% of fatalities in Ghana [12].

Protozoan parasites have a severe effect on both pregnant mothers and their developing foetus and complications such as stillbirth, anaemia, neonatal sepsis, neonatal death, and low birth weight are observed irrespective of the measures being adopted to control malaria [13]. However, the impact of parasitic infections on a mother or child is determined by the mother's natural immunity, the kind of parasite, and the parasitic burden. The WHO recommends that endemic nations provide regular treatment (deworming) to at-risk populations such as nursery and school-age babies, women of reproductive age, and adults in certain high-risk occupations in order to reduce and eventually eliminate intestinal parasitic infection (IPI)-related morbidity [14].

Blood and intestinal parasite infection loads should be monitored on a regular basis to avoid the consequences listed above. In immunocompromised individuals, *Toxoplasma gondii* infection can result in severe clinical consequences [15]. Toxoplasma infections need to be screened at antenatal care clinics to further prevent congenital transmission to the developing foetus [16]. Seroprevalence of anti-*T. gondii* IgG was 51.2% among pregnant women in Accra [17]. Studies in other coastal parts of Ghana indicate the presence of toxoplasmosis in the country, but not much work has been done in the northern part and middle belt of the country where adequate health facilities are limited [18].

The effect of intestinal and blood protozoan infections could have mild to severe effects on the mother and the unborn baby [19]. Even though research on intestinal protozoa has been done, it has primarily focused on schoolchildren rather than adults, particularly pregnant women. Since there is a detrimental effect of these parasites among pregnant women, this study attempts to determine how prevalent these protozoans are among pregnant women. It is therefore imperative that protozoan prevalence among pregnant women in Kumasi be conducted to assess the burden of infection and the potential risk factors that may be associated with the transmission of the infection.

## 2 Materials and methods

### 2.1 Study site/design

The pilot cross-sectional study was conducted at Tafo government hospital within the Kumasi metropolis (Manhyia North Sub-Metropolis to be precise) from November 2016 to January 2017. Kumasi Metropolis is the second-largest city in Ghana and the capital town of the Ashanti Region. Kumasi lies on latitude 6°35″ and 6°40″ and longitude 1°30″ and 1°35″ E. It has a landmass of 254 km and an average temperature between 22 °C and 31 °C. The population of pregnant women receiving ante-natal care ranges from nine hundred and fifty (950) to about one thousand two hundred (1200) within a month.

### 2.2 Map for study site

See Figure 1.

**Figure 1:**
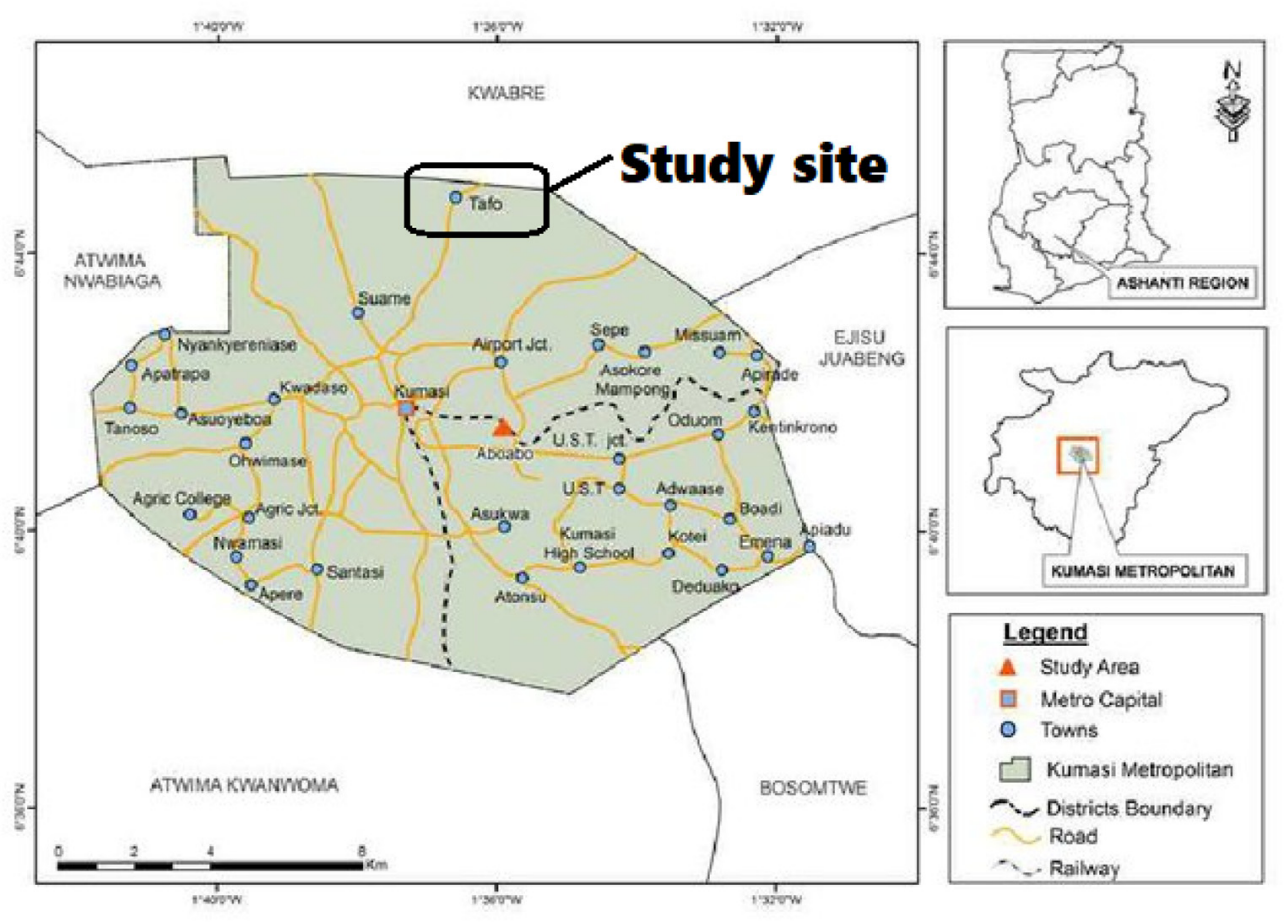
: A map study site Tafo a town in Kumasi. Adapted from Mensah *et al.* (2013) [20].

### 2.3 Study population

Participants for the study were pregnant women visiting the Antenatal Care (ANC) at Tafo Government hospital in Kumasi Metropolis from November 2016 to January 2017. The sample size is one hundred and fifty (150). The sample size was calculated from the equation below; Sample size = (Z^2^ (P) (1 − P))/E ^2^, where: Z = the number relating to the degree of confidence you wish to have in the result. The standard score for the confidence interval of 95% is 1.96. P = Previous study’s prevalence as obtained from Ayi et al. (2009), P = 92.5% is (0.925). E = Error was calculated at 5% (0.05). Therefore: Sample size = (1.96)^2^ (0.925) (1–0.925)/(0.05) ^2^ = 106.6 ≈ 110. However, to accommodate a nonresponse rate of 100% and stronger statistical power and effect size, the samples were projected to 150 participants for the study. The excluded participants were expectant women, those under 18 and over 49 years old, who were in critical condition and needed medical treatment. Qualified to participate in the research were all pregnant women who fulfilled the eligibility requirements and consented after the purpose and goals had been clarified to them.

### Ethical approval

Ethical clearance for the study was obtained from the Committee on Human Research, Publication and Ethics (CHRPE) at the KNUST School of Medical Sciences/Komfo Anokye Teaching Hospital in Kumasi Ghana (CHRPE/AP/299/17)**.** Permission was also given by the Medical Director and the head of the laboratory at the hospital. Written informed consent was also obtained from study participants who were adequately informed of the procedures, nature, risk, and the purpose of the study. Qualified patients were made to fill or thumbprint a consent form with the help of the research team before their wards were recruited for the study according to the Helsinki declaration [21].

### 2.4 Sample processing and examination

About 4g of stool specimens were collected from each of the participants.

### 2.5 Stool preparation for microscopic examination of parasites

#### 2.5.1 Wet mount

A drop of fresh physiological saline and iodine (Baxter International Inc., USA) were placed at the ends of a clean 26 × 76 mm microscope slide (Avantik, USA). About 2mg of stool was picked with an applicator stick and mixed with saline to obtain a smooth preparation. The same amount of stool was picked again from the iodine end on the same microscope slide and mixed well to smooth preparation. Each preparation was then covered with a coverslip. Using 10x and 40x objectives, the wet preparations were examined for trophozoite larvae or cysts of intestinal parasites.

#### 2.5.2 Faecal protozoans concentration method

About 1g of stool sample was transferred into a well-labeled 14 ml falcon tube (352070 Corning, USA). 7 ml of sodium acetate, acetic acid, formalin solution (Para-Fix™: SAF, MCC, USA) were poured into the falcon tube containing the stool. The tubes were capped and mixed well by shaking them. The content of the stool was then sieved through an 80 μm spore net sieve (Wire cloth company, USA) with the aid of a funnel into a beaker. The content was then transferred into a new labeled falcon tube. The filtrate in the falcon tube was spun at 2000 rpm for a minute. The supernatant was discarded and 7 ml of physiological saline was added. 3 ml of diethyl ether was also added to reach the 10 ml mark of the tube. The mixture was well capped and mixed using a vortex mixer (Scientific Industries, Inc., USA) for 15 s. The tube was gently uncapped to release gas from the tube. The mixture was spun again at a speed of 3000 rpm for 1 min. The contents of the tube were discarded leaving the sediment, from which small portions were transferred onto a microscope slide and coverslip placed on it for microscopic examination. Another portion of the sediment was smeared on a microscope slide for staining to detect oocytes of the *Cryptosporidium parvum* [22].

#### 2.5.3 Staining of stool smears for detection of intestinal protozoans

Smears were made from the concentration sediment. The smears were air-dried and fixed in methanol for 2–3 min. The slides were then stained with unheated carbol fuchsin for 15 min. The stains were washed off with water. 1% acid alcohol was used to decolorize for 10–15 s and washed off with water. Methylene blue was used to counterstain the slides for about 30 s and washed off with water. Slides were air-dried and examined under 40x and 100x objective lenses for the detection of intestinal protozoans [23].

#### 2.5.4 Parasite identification and stigmatization of hemoglobin level among participants

Venous blood was taken for the detection of blood protozoans in the study participants. Three millilitres of blood was taken from each participant by an aseptic technique into an EDTA (Ethylenediaminetetraacetic acid) tube using a sterile hypodermic syringe and needle.

Thick and thin film preparation was made on the same slides and stained in Giemsa. The thick films were prepared to detect the parasite and parasite density (quantification). The thin films were done for parasite species identification and quantification.

The Plasmodium trophozoites seen were counted as well as the WBC (white blood cells) using two hand tally counters. The examination was continued until about 100 fields were examined and the parasites and WBC counted separately. The density of the parasites was calculated from the formula: (no of parasite counted)/(no of WBC counted) x8000/μL, where 8000 is the standard WBC count. The hemoglobin concentration was measured using an automated hematological analyzer (Sysmex Automated Hematology Analyzer, Kobe, Japan, XP-300).

#### 2.5.5 Enzyme-linked immunosorbent assay (ELISA), for *T. gondii*

The remaining blood of about 2.5 ml of the EDTA was spun and the plasma (supernatant) was collected using a pipette into a labeled sterile cryotube. The plasma was stored at −80 °C temperature until used. The antibodies (IgM and IgG) of *T. gondii* in the plasma samples were qualitatively detected using a commercial kit (Fortress Diagnostic Limited, Antrim, United Kingdom), following the instructions of the manufacturer [24].

#### 2.5.6 Data analysis

The data obtained were analysed using the Statistical Package for Social Sciences (SPSS) version 20 for Windows as well as the Graph Pad Prism version 6 statistical software. Descriptive analysis of variables was conducted using frequency analysis, whereas the risk factors of intestinal protozoan infection were analysed using chi-square for the categorized variables. Fisher’s exact test was used to determine the population at risk of diseases. A comparison of hemoglobin medians was done between the negatives and positives. Statistical significance was accepted at P < 0.05 for all comparisons.

## 3 Results

### 3.1 Socio-demographic characteristics of participants

This study constituted a total of 150 pregnant women. The age range of the participants was between 18 and 40 years with a mean age of 27.8. Higher proportions of the study participants were within the age range 20–29 years (58.0%) and the majority of them (82.2%) had not experienced a stillbirth or spontaneous abortion before. Regarding educational level, 79 (52.6%) had only basic education and about 79.3% are informally employed. More than half of the pregnant women 115 (76.7%) resided in an urban setting and the majority of the study participants (44.7%) were in their 2^nd^ Trimester (Table 1).

**Table 1:** Socio-Demographic Characteristics of study participants.

Variable	Frequency (n = 150)	Percentage (%)
**Age(years) (mean ± SD)**	27.83 ± 5.29	
**Age group(years)**		
<19	10	6.7
20–29	87	58.0
30–39	50	33.3
>40	3	2.0
**Educational level**		
No education	13	8.7
Basic education	79	52.6
Secondary education	43	28.7
Tertiary education	15	10.0
**Occupational status**		
None	2	1.4
Informally employed	119	79.3
Formally employed	29	19.3
**Residency**		
Urban	115	76.7
Urban-slum	7	4.6
Rural	28	18.7
**Gestational age**		
1st Trimester	48	32.0
2nd Trimester	67	44.7
3rd Trimester	35	23.3
**Gravida**		
Primigravida	40	26.7
Secundigravida	47	31.3
Tertigravida	41	27.3
Quartigravida	20	13.3
Quintigravida	2	1.4
**Stillbirths/spontaneous abortion**		
Never	123	82.0
Once	20	13.3
Twice	7	4.7

SD = Standard Deviation.

**Figure 2:**
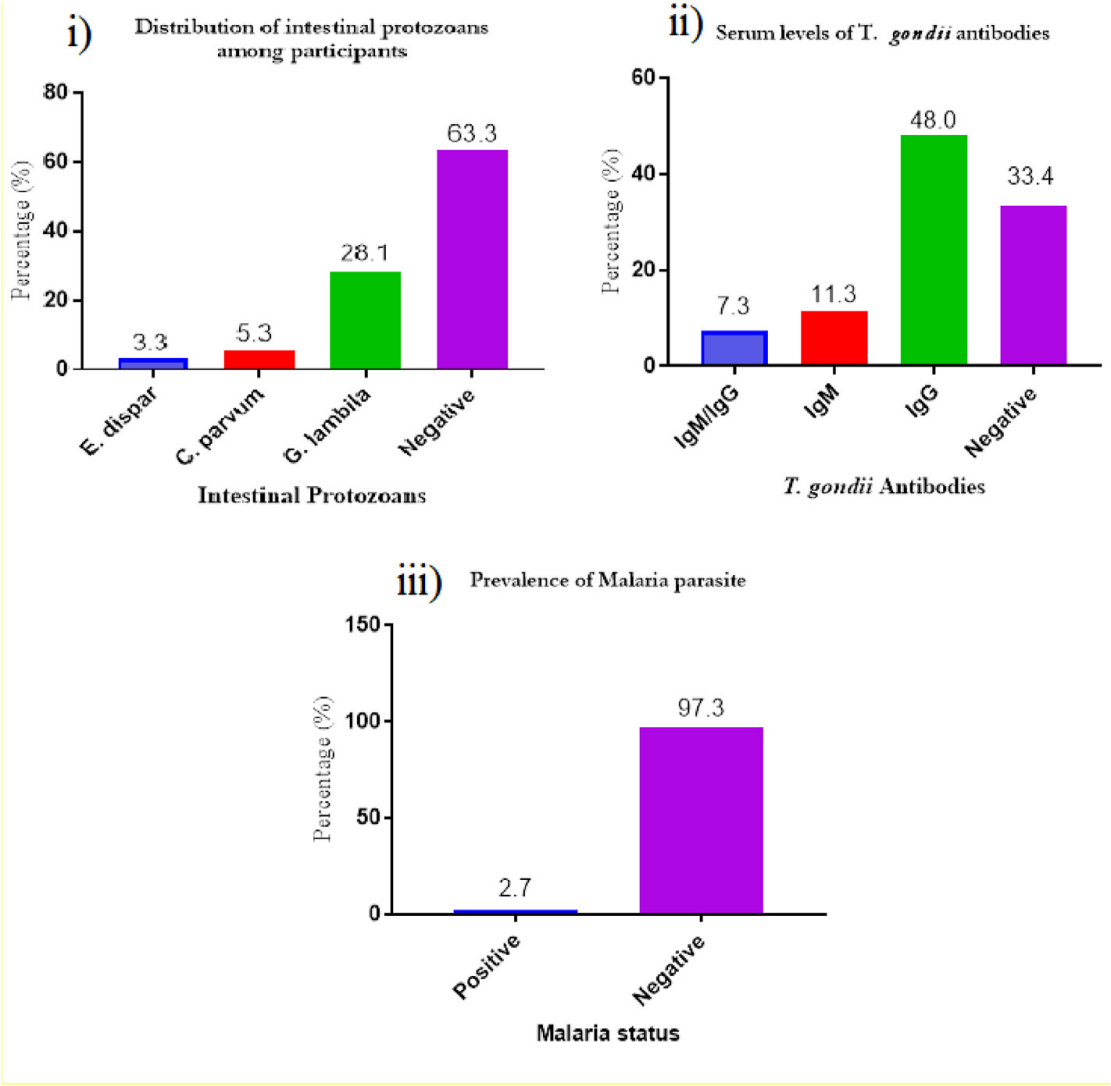
i) Distribution of intestinal protozoans, ii) Serum levels of *T. gondii* antibodies among study participants, iii) malaria parasite infection.

From Figure 2; i) Out of the 150 participants examined for the presence of intestinal parasites, 28.1% were infected with *Giardia lamblia*, 5.3% were infected with *C. parvum* and 3.3% were infected with *Entamoeba dispar*. The overall prevalence of intestinal protozoan parasites was 36.7%. ii), *T. gondii* seroprevalence rates were 11.3% for IgM, 48.0 for IgG, and 7.3% for both IgM/IgG. iii), Only four out of the 150 participants were positive for Plasmodium infection. This observation was for the microscopic examination of both thin and thick films as well as the rapid diagnostic test (RDT). *Plasmodium falciparum* was the only species identified. The mean parasite loads of 2.7% positives were 2060/uL.

### 3.2 Knowledge of participants on intestinal parasites

The majority of the participants (98.7%) were unaware of the concept of intestinal protozoans, even though 30% of the participants used various kinds of dewormers as prophylaxis. There was a significant association between those who take dewormer as prophylaxis and intestinal protozoan infection status. Most of the participants (97.3%) knew of malaria infection, however, almost all participants (99.3%) were unaware of the *T. gondii* parasite. The results are illustrated in Table 2.

**Table 2:** Knowledge of Participants on Intestinal parasites.

	Total	Positive	Negative	P-value
**Knowledge of protozoan disease**				0.694
Yes	2 (1.3%)	1 (1.8%)	1 (1.1%)	
No	148 (98.7%)	54 (98.2%)	94 (98.9%)	
**Dewormer as prophylaxis**				**0.042**
Yes	45 (30.0%)	11 (20.0%)	34 (35.8%)	
No	105 (70.0%)	44 (80.0%)	61 (64.2%)	
**Knowledge of malaria**				0.737
Yes	146 (97.3%)	4 (100.0%)	142 (97.3%)	
No	4 (2.7%)	0 (0.0%)	4 (2.7%)	
**Knowledge of toxoplasmosis**				0.335
Yes	1 (0.7%)	1 (1.3%)	0 (0.0%)	
No	149 (99.3%)	77 (98.7%)	72 (100.0%)	

P-value<0.05 = statistically significant.

### 3.3 Predisposing risk factors of intestinal protozoan infections among study participants

Information on lifestyle characteristics such as dietary intake, source of water, type of toilet facility used, and having a cat as a pet that disposes one to intestinal protozoans and *T. gondii* infections were obtained from the study participants. There was no significant association between the source of water (p = 0.655) or food (p = 0.411) taken by any of the study participants. The type (p = 0.977) or state (p = 0.575) in which meat was consumed was also not associated with the infestation of intestinal protozoa. However, there was a significant association between the state of vegetables the study participants consumed and the prevalence of intestinal infections (p = 0.004). Those who consume fresh and raw vegetables are at high risk of acquiring intestinal infections compared to those that took in cooked vegetables (OR = 0.32, CI = 0.12–0.86) as shown in Table 2. There was no statistically significant association between the predisposing risk factors and the *T. gondii* infection among the participants in Table 3 (see Table 4).

**Table 3:** Predisposing risk factors of intestinal protozoan infections.

Variable	Intestinal protozoan infections			
	Positive	Negative	P-value.	OR (CI = 95%)
**Source of water**			0.655	
Pipe borne	34 (61.8%)	65 (68.4%)		1 (referent)
Well	10 (18.2%)	16 (16.9%)		1.20 (0.49–2.92)
Borehole	11 (20.0%)	14 (14.7%)		1.50 (0.62–3.67)
**Source of food**			**0.411**	
Food vendors	1 (1.8%)	2 (2.1%)		1 (referent)
Self-cooking	5 (9.1%)	16 (16.8%)		0.63 (0.04–8.44)
Food vendors/self-cooking	49 (89.1%)	77 (81.1%)		1.27 (0.11–14.42)
**Type of toilet**			**0.292**	
Pit latrine	5 (9.1%)	3 (3.2%)		1 (referent)
Public toilet	22 (40.0%)	39 (41.0%)		0.34 (0.07–1.55)
Water closet	28 (50.9%)	53 (55.8%)		0.32 (0.07–1.43)
**State of meat eaten**			**0.575**	
Cooked till soft	16 (29.1%)	28 (29.5%)		0.79 (0.34–1.81)
cooked tough	21 (38.2%)	29 (30.5%)		1 (referent)
cooked tough/soft	18 (32.7%)	38 (40.0%)		0.65 (0.29–1.45)
**Type of meat eaten**			**0.977**	
Beef, chevon, mutton	24 (43.6%)	42 (44.2%)		1 (referent)
Beef, chevon, pork	24 (43.6%)	40 (42.1%)		1.05 (0.51–2.14)
Beef, mutton, pork	7 (12.8%)	13 (13.7%)		0.94 (0.33–2.69)
**State of vegetable eaten**			**0.004**	
Fresh and raw	46 (83.6%)	54 (57.5%)		1 (referent)
Cooked	6 (10.9%)	22 (23.4%)		0.32 (0.12–0.86)
Fresh/cooked	3 (5.5%)	18 (19.1%)		0.39 (0.14–1.07)
**Cat as pet**			**0.053**	
Yes	15 (27.3%)	41 (43.2%)		1 (referent)
No	40 (72.7%)	54 (56.8%)		0.89 (0.40–1.98)

OR = Odd ratio, CI-Confidence level, p-value<0.05 = statistically significant.

**Table 4:** Predisposing risk factors of *Toxoplasma gondii* infection.

Variable	*Toxoplasma gondii* infections			
	Positive	Negative	P-value.	OR (CI = 95%)
**Source of water**			0.374	
Pipe borne	48 (61.5%)	51 (70.8%)		1 (referent)
Well	14 (17.9%)	12 (16.7%)		1.24 (0.52–2.94)
Borehole	16 (20.6%)	9 (12.5%)		1.89 (0.76–4.68%)
**Source of food**			**0.808**	
Food vendors	1 (1.3%)	2 (2.8%)		1 (referent)
Self-cooking	11 (14.1%)	10 (13.9%)		2.20 (0.17–28.16)
Food vendors/self-cooking	66 (84.6%)	60 (83.4%)		2.20 (0.19–24.90)
**Type of toilet**			**0.208**	
Pit latrine	4 (5.2%)	4 (5.6%)		1 (referent)
Public toilet	37 (47.4%)	24 (33.3%)		1.54 (0.35–6.76)
Water closet	37 (47.4%)	44 (61.1%)		0.84 (0.20–3.60)
**State of meat eaten**			**0.321**	
Cooked till soft	20 (26.3%)	22 (30.6%)		0.61 (0.26–1.39)
cooked tough	30 (39.5%)	20 (27.8%)		1 (referent)
cooked tough/soft	26 (34.2%)	30 (41.7%)		0.56 (0.27–1.15)
**Type of meat eaten**			**0.729**	
Beef, chevon, mutton	33 (42.3%)	33 (45.8%)		1 (referent)
Beef, chevon, pork	33 (42.3%)	26 (36.1%)		1.27 (0.62–2.57)
Beef, mutton, pork	12 (15.4%)	13 (18.1%)		0.92 (0.62–4.26)
**State of vegetable eaten**			**0.611**	
Fresh and raw	50 (64.1%)	50 (69.4%)		1 (referent)
Cooked	15 (19.2%)	14 (19.4%)		0.91 (0.47–2.45)
Fresh/cooked	13 (16.7%)	8 (11.2%)		0.97 (0.62–4.26)
**Cat as pet**			**0.706**	
Yes	30 (38.5%)	46 (36.1%)		1 (referent)
No	48 (61.5%)	46 (63.9%)		0.90 (0.47–1.76)

OR = Odd ratio, CI-Confidence level, p-value<0.05 = statistically significant.

**Figure 3:**
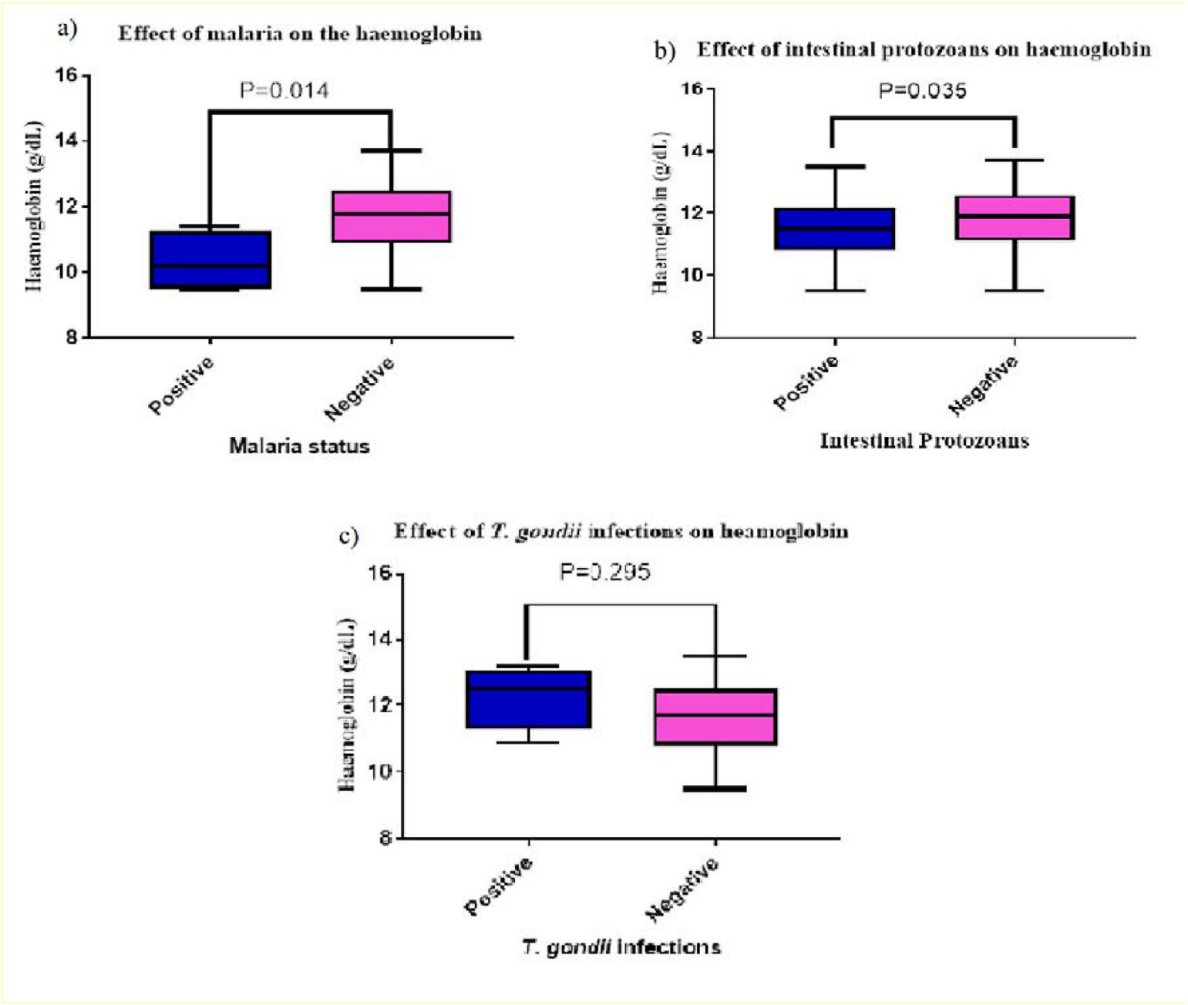
Haemoglobin Concentration in Stratified a)Malaria Infections, b) Stratified intestinal protozoan Infections, and c) *T. gondii* Infections among Participants.

From Figure 3, Participants with malaria infection had low haemoglobin compared with those without malaria (p = 0.014). Participants with intestinal protozoan infection had low haemoglobin levels compared to those without infection, (p = 0.035). However, there was no significant difference (p = 0.295) between the Hb levels in individuals with antibodies to *T. gondii* compared to those without antibodies.

## 4 Discussion

Infections such as intestinal protozoans are mostly found in the tropical and subtropical regions of Sub-Saharan countries that harbor environmental and socio-cultural factors that aid in parasitic transmission [25]. The severity of parasite infection among pregnant women and their developing foetuses is partly due to both the compromised health status and nutritional status of the women [26]. Nonetheless, the severity of the effects depends on several factors such as the parasite species involved, the load of the parasite, the immunity of the pregnant woman, and the presence of co-existing disease conditions [26]. This study was conducted among pregnant women in Kumasi to assess the prevalence of intestinal and blood protozoan infections and the risk factors associated with these infections.

In this present study, a prevalence of 36.7%, intestinal protozoan infection was described morphologically among the study participants. The dramatic prevalence of intestinal protozoan infections described in this study is similar to the reports from other countries such as Mexico (38.2%) that have similar environmental, physical, and socioeconomic factors with Ghana [27].

*G. lamblia* infection recorded a prevalence of 28.1%, making it the most important intestinal protozoa in this study. A prevalence rate of 12.17% was reported by Walana (2014) among school children in Kumasi. The environmental and unavailability of potable water were causes of infection among the school children in Kumasi [28]. It is therefore not surprising when consumption of raw fruits and vegetables, environmental hygiene, and source of drinking water were identified as factors that expose these pregnant women to parasitic infections [29].

The second most predominant intestinal protozoa in this study were *C. parvum* with a prevalence of 5.3%. Immunocompromised individuals such as pregnant women, very old, and young people experience severe forms of cryptosporidiosis which include crampy abdominal pain, profuse diarrhea with malabsorption, fatigue, nausea, and vomiting [30]. A prevalence rate of 8.5% was reported among school children in Kumasi [28]. Aly and Mostafa (2010), reported a prevalence rate of 0.6% in the Saudi Arabia Kingdom. The lower prevalence in the Saudi Kingdom was attributed to a good source of drinking water and proper hygiene practices [31].

*E. dispar* infection has been reported throughout the world at a rate that parallels the level of personal hygiene and sanitation within a community [32]. A prevalence rate of 0.21% of *Entamoeba histolytica* infection was reported by Walana *et al.* (2014) in Kumasi among school children which is lower than was obtained in this present study. The prevalence rate of 3.3% obtained in this study was high, possibly due to the consumption of raw fruits and vegetables.

The high prevalence of intestinal protozoa infection exceeding the 20% [33] limit could be attributed to secondary factors such as poverty, improper washing of hands, and the source of water consumed such as boreholes and wells, which are the favorable conditions for parasite prevalence in Ghana [34]. Even though the majority of the study participants lived in urban settings, they lacked knowledge about protozoa infection. Greater than half of the pregnant women buy or cook food, wash food items with only water before consuming. Moreover, most of the participants have no idea how food vendors prepare food items for selling, how to properly handle the meat they bought from the market, which are all routine activities of these women, therefore, predisposing them to intestinal protozoa infection. Although deworming confers a secondary defense against parasitic infections through adaptive mechanisms, the majority of pregnant women failed to take dewormers before pregnancy as most of them had no knowledge on intestinal and blood protozoans [35].

A tropical disease such as malaria has been reported for its major impact on reproductive health [36]. Thus, malaria remains a significant tropical parasitic infection in pregnancy that contributes importantly to anemia and the cycle of retarded growth and development [27, 37]. An estimated malaria prevalence of 2.7% was reported among the study participants. This was not surprising because the majority of the participants knew about malaria disease. In a systematic review and meta-analysis by Chico *et al.*, in sub-Saharan Africa, the pooled prevalence of peripheral and placental malaria was 38.2% and 39.9%, respectively across data regions that included Ghana [38]. The low malaria prevalence in this study is a good indication of the success of the national control programs underway in Ghana that contributed to 90% of the participants taking malaria prophylaxis [39]. In this present study, Hb levels were lower in infected malaria individuals compared to the uninfected. The mean Hb of 9.5 g/dl could be an indication of red cell hemolysis that could lead to anaemic conditions. Independent of the etiology, protozoan infections have been reported to be associated with reduced Hb among pregnant women [40].

The study reported high serum levels of *T. gondii* antibodies among pregnant women. *T. gondii* seroprevalence rates were 11.3% for IgM, 48.0% for IgG, and 7.3% for both IgM/IgG. Another study in patients of an average age of 30.2 years visiting the Korle-Bu Teaching Hospital in Accra reported a seroprevalence rate of 92.5 % [41]. Furthermore, a population-based study in coastal Ghana reported a seroprevalence of 85% of *T. gondii* [24]. The higher prevalence reported in this study and other studies across the globe indicated that *T. gondii* infections represent one of the most ubiquitous intracellular protozoan parasites with worldwide distribution. It has been estimated that one-third of the world's population has been exposed to *T. gondii* [42]. However, most infections are asymptomatic in some individuals; the parasite can become widely disseminated causing severe clinical signs in immunocompromised individuals. There was 11.3% of participants positive to the serum of *T. gondii* antibodies by IgM detection in this study. A positive Toxoplasma immunoglobulin M (IgM) result is often interpreted as a marker of an acute infection contrary to the IgG dye test, which is considered the chronic infection state for *T. gondii* [43]. Despite the high seroprevalence rate of T. *gondii* antibodies recorded in this present study and other data regions, the majority of pregnant women included in this study had no knowledge about the disease and had never had a screening for *T. gondii* antibodies. Studies have indicated that humans become infected by ingesting food or water contaminated with oocysts shed by cats; congenitally by the trans-placental transmission of tachyzoites; by eating undercooked or raw meat containing tissue cysts [44, 45]. The infection of *T. gondii* and the consumption of meat did not show any significant association, yet studies conducted by Arko-Mensah et al., (2000) reported that most farm animals are infected with the bradyzoites and tachyzoites of *T. gondii* [46]. In this study, most of the pregnant women buy cooked food or washed food items with only water before consuming. Most of these pregnant women do not have any idea how food vendors prepare food items before selling and how well meat products are treated before consumption, which are predisposing factors to protozoa infection like *T. gondii*. The tachyzoites of *T. gondii* parasites attack nucleated cells such as leukocytes and macrophages and by penetrating the cells after ingestion [47]. The tachyzoites released from the burst of macrophages are then distributed into muscle tissues and organs such as the liver, spleen, eye, and brain through the bloodstream. However, it does not cause anaemia because it does not attack matured erythrocytes [47]. This was the case in this study as there was no significant difference between the *T. gondii* infected individuals and the uninfected concerning hemoglobin level.

### 4.1 Limitations of the study

This study is limited by its sample size due to insufficient funds and the limited time when recruiting the study participants and so the sample size was not enough to potentially identify any significant risk factor. Therefore this study seeks to serve as a pilot study for a larger study. This study can also serve as a blueprint for larger studies including following up mothers and children to obtain a better picture of a more well-informed conclusion about these important protozoan infections. The prevalence of intestinal parasite infection in this investigation was based on a single fecal sample rather than the optimum three consecutive samples. Furthermore, despite the fact that microscopy was used to detect parasite infections, the method's limited sensitivity may have resulted in a significant number of missed cases, especially in the presence of low-intensity infections. Additional research might include nutritional evaluation and infection density, which were not accessible in this study. Most participants may have been on anti-malarial drugs which they do not remember. Since PCR test was not done, *E*. *dispar* was noted instead of *E. histolytica.*

## 5 Conclusion

This present study showed an intestinal protozoa prevalence of 36.8% among pregnant women in Tafo Hospital, Kumasi. The most prevalent protozoans were *G. lamblia* with a rate of 28.1% and *C. parvum* with a prevalence of 5.3%. Those who eat inadequately cleaned fresh and raw vegetables were at a higher risk of intestinal protozoan infections than those who take in the cooked vegetable. An 11.3% IgM T. *gondii* antibodies indicate that few of the infections by *Toxoplasmosis gondii* were in the acute stage and a low prevalence rate of 2.7% of malaria among pregnant women. Environmental hygiene should be improved and relevant agencies should educate the public on the transmission of intestinal parasite infections. Screening of both blood and intestinal protozoans should be routinely done for pregnant women visiting the ANC. The population at risk should be educated on the adverse effects intestinal and blood protozoan infections have on pregnancy.

## Declarations

### Author contribution statement

Emmanuel Amaniampong Atakorah: Conceived and designed the experiments; Performed the experiments; Contributed reagents, materials, analysis tools or data; Wrote the paper.

Bright Oppong Afranie: Performed the experiments; Analyzed and interpreted the data; Wrote the paper.

Kwabena Darko Addy and Bright Afranie Okyere: Analyzed and interpreted the data; Contributed reagents, materials, analysis tools or data; Wrote the paper.

Ama Darkoaa Sarfo: Performed the experiments; Contributed reagents, materials, analysis tools or data; Wrote the paper.

### Funding statement

This research did not receive any specific grant from funding agencies in the public, commercial, or not-for-profit sectors.

### Data availability statement

Data will be made available on request.

### Declaration of interests statement

The authors declare no conflict of interest.

### Additional information

No additional information is available for this paper.

## Figures and Tables

**Figure:**
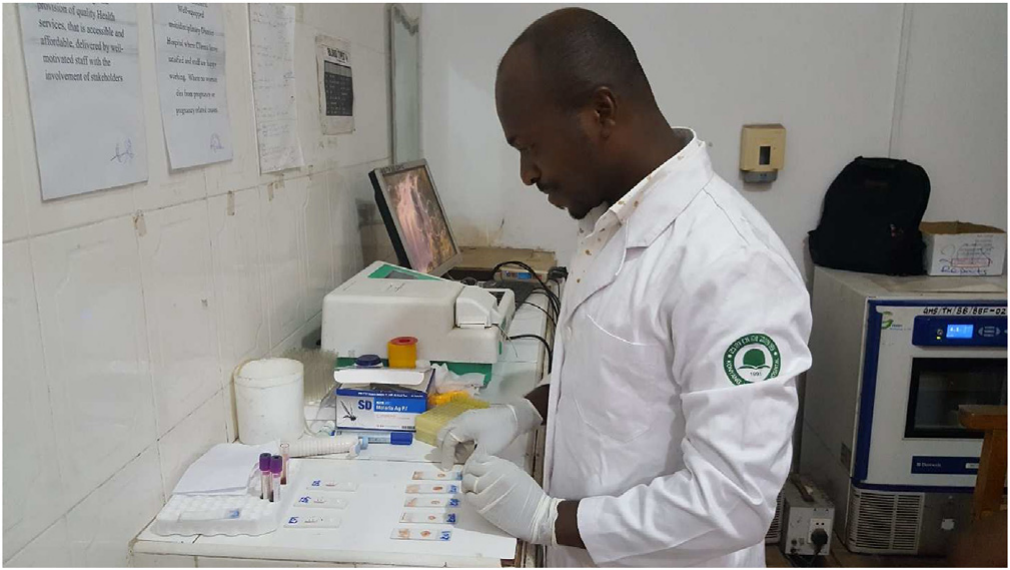
Preparation of smears

**Figure:**
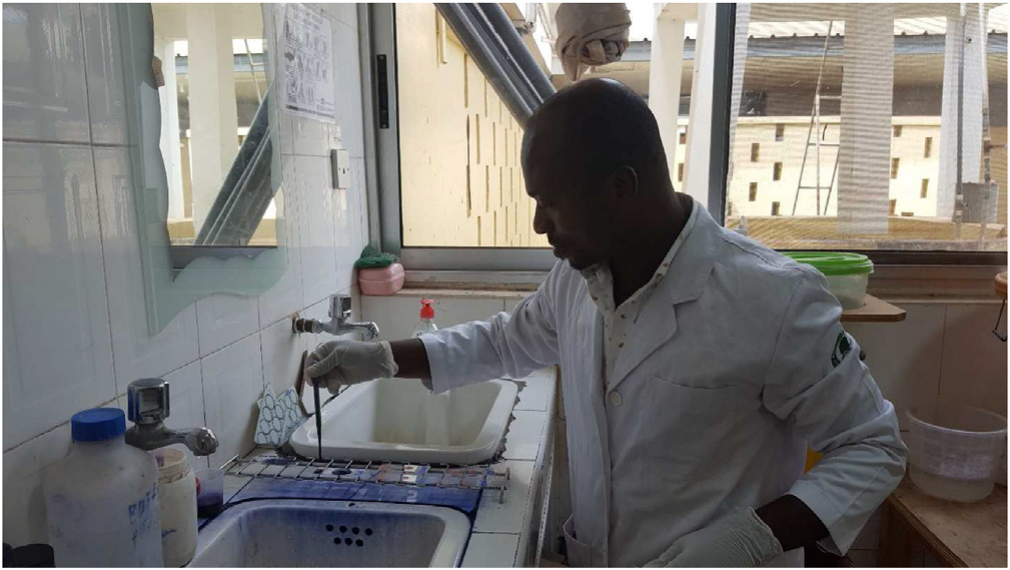
Staining of prepared smears

**Figure:**
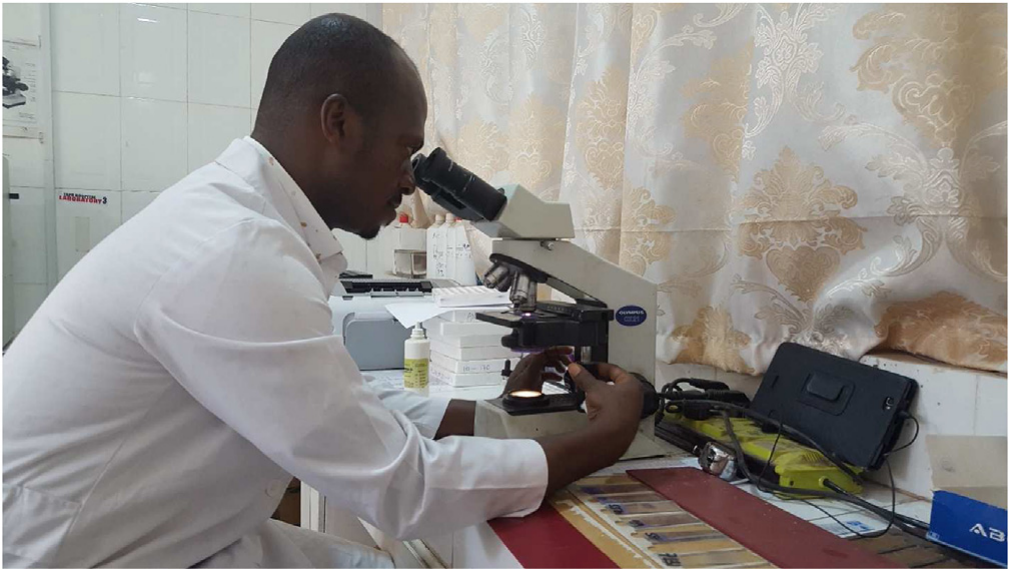
Examination of Haemoglobin concentrations

**Figure:**
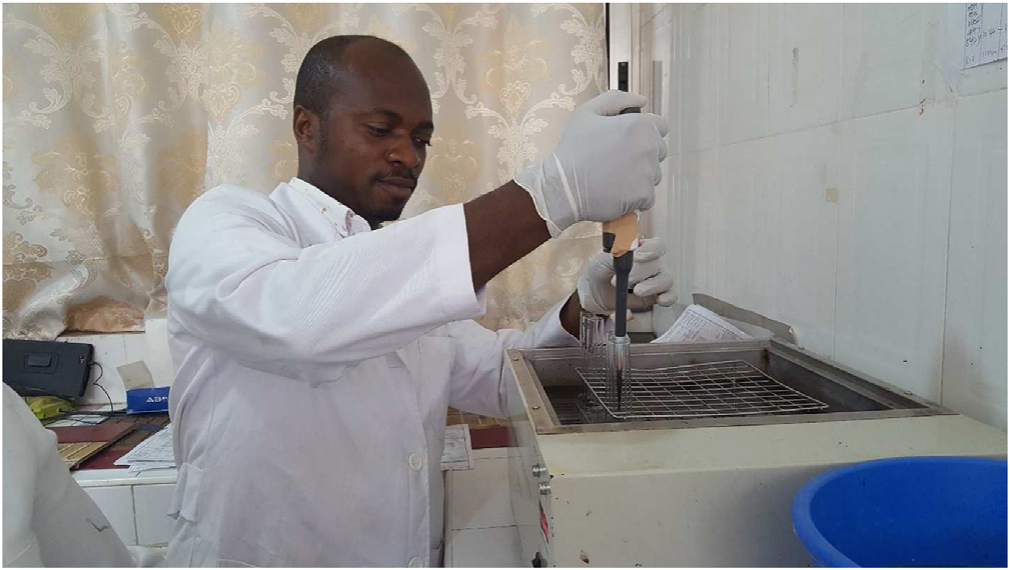
Preparation of smears

**Figure:**
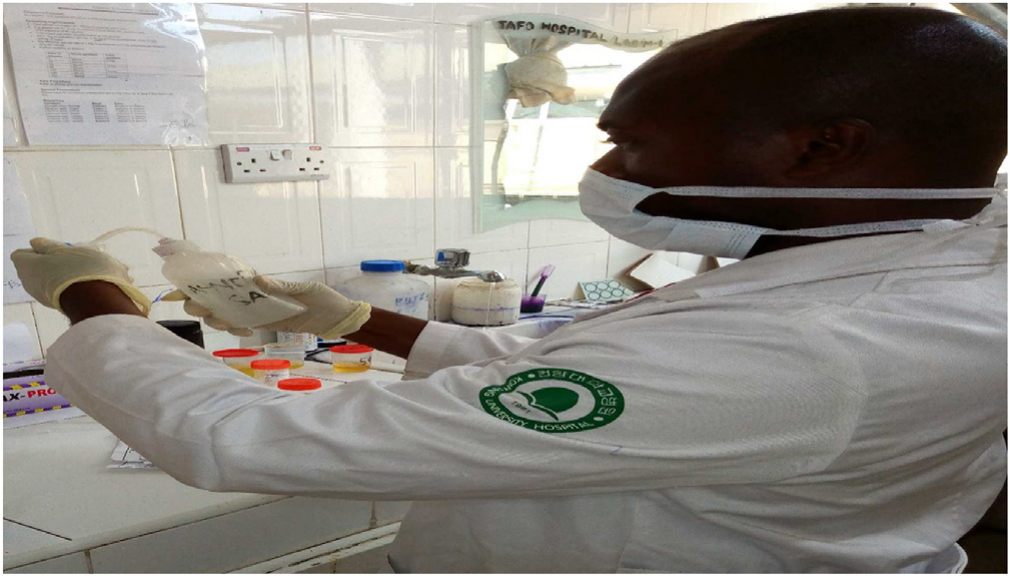
Haemoglobin Concentration in Stratified a)Malaria Infections, b) Stratified intestinal protozoan Infections, and c) *T. gondii* Infections among Participants.

**Figure:**
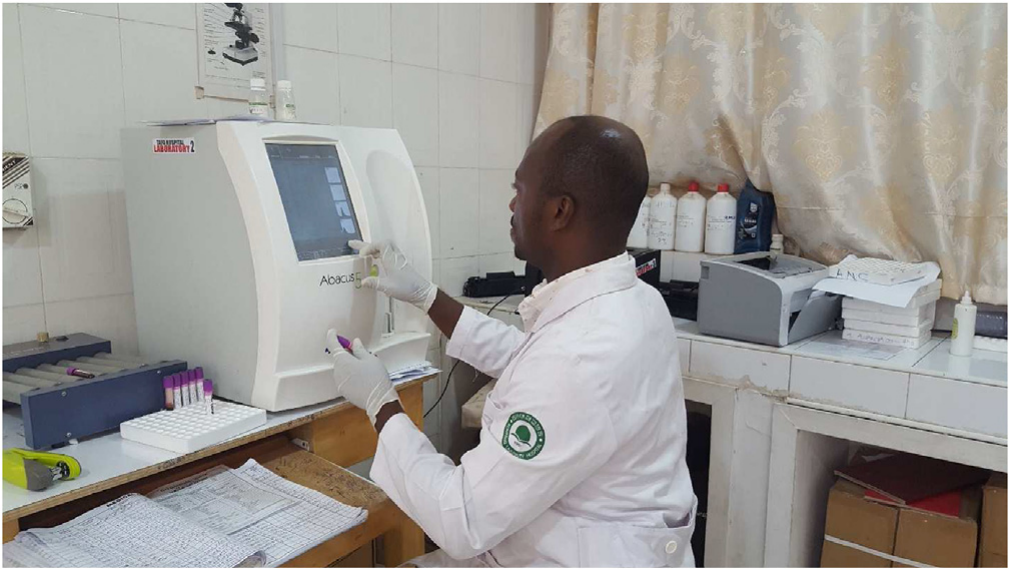
Examination of stained slides under microscope

## Appendix

Appendix S1: images of preparation of smear, staining of prepared smear, examination of stained slides under the microscope, preparation of plasma, preparation of stool sample for observation, examination of haemoglobin concentration.
